# Clinical Value of ^18^F-FDG PET/CT Scan and Cytokine Profiles in Secondary Hemophagocytic Lymphohistiocytosis in Idiopathic Inflammatory Myopathy Patients: A Pilot Study

**DOI:** 10.3389/fimmu.2021.745211

**Published:** 2021-11-18

**Authors:** Junyu Liang, Heng Cao, Bowen Wu, Yinuo Liu, Ye He, Bei Xu, Yiduo Sun, Bingjue Ye, Jin Lin

**Affiliations:** ^1^ Department of Rheumatology, The First Affiliated Hospital, Zhejiang University School of Medicine, Hangzhou, China; ^2^ Department of Hematology, The First Affiliated Hospital, Zhejiang University School of Medicine, Hangzhou, China; ^3^ PET Center, The First Affiliated Hospital, Zhejiang University School of Medicine, Hangzhou, China; ^4^ Department of Respiratory Diseases, The First Affiliated Hospital, Zhejiang University School of Medicine, Hangzhou, China

**Keywords:** idiopathic inflammatory myopathy, secondary hemophagocytic lymphohistiocytosis, PET/CT, cytokine profile, dermatomyositis, polymyositis

## Abstract

**Background:**

Secondary hemophagocytic lymphohistiocytosis (sHLH) is a rare but fatal complication in idiopathic inflammatory myopathy (IIM) patients. The clinical value of radiological manifestations and serum cytokines remain unknown in this systemic crisis. This study aims to investigate the clinical value of PET/CT scan and cytokine profiles in predicting and understanding sHLH in IIM patients.

**Methods:**

Adult IIM patients who were admitted to the four divisions of the First Affiliated Hospital, Zhejiang University School of Medicine (FAHZJU) from January 1, 2017 to December 31, 2020 were reviewed. PET/CT scan, cytokine profiles, and other factors of patients who met the inclusion and exclusion criteria were collected and analyzed.

**Results:**

Sixty-nine out of 352 IIM patients were finally enrolled into the study. Ten patients developed sHLH and 70.0% of them died within 6 months. After false discovery rate (FDR) correction and multivariate logistic regression analysis, increased serum interferon (IFN)-γ level (*p* = 0.017), higher spleen mean standard uptake value (SUVmean, *p* = 0.035), and positivity of anti-MDA5 antibody (*p* = 0.049) were found to be significantly correlated with development of sHLH in IIM patients. The combination of serum IFN-γ, spleen SUVmean, and anti-MDA5 antibody found a balanced and satisfying predictor with a cutoff value of 0.047 and AUC of 0.946. A moderate correlation was identified between ferritin and spleen SUVmean (*p* = 0.001, *r* = 0.380) as well as serum IFN-γ(*p* = 0.001, *r* = 0.398). Before FDR correction, higher bilateral lung SUVmean (*p* = 0.034) and higher colon/rectum SUVmean (*p* = 0.013) were also observed in IIM patients who developed sHLH. By narrowing down to IIM patients with sHLH, anti-MDA5-antibody-positive DM patients tended to suffer from unfavorable outcome (*p* = 0.004) in Kaplan–Meier survival analysis.

**Conclusion:**

Increased serum level of IFN-γ, elevated splenic FDG uptake, and positivity of anti-MDA5 antibody were significantly correlated with development of sHLH in IIM patients. Lung and lower digestive tract might also be affected due to systemic immune activation in IIM patients with sHLH. In addition, splenic FDG uptake, in combination with serum IFN-γand anti-MDA5 antibody, was found valuable in predicting development of sHLH in IIM patients. Among IIM patients with sHLH, anti-MDA5-antibody-positive DM patients showed higher tendency for unfavorable outcome.

## Introduction

Hemophagocytic lymphohistiocytosis (HLH) is a rare but life-threatening disorder featuring multiple clinical manifestations including continuous fever, cytopenia, excessive cytokine production, splenomegaly, hyperferritinemia, coagulopathy, and multiorgan failure (1, 2), with an estimated annual incidence of 1 per 800,000 adults and 1 to 10 per 1 million children in western Europe and the USA (2). In terms of pathophysiological mechanism, HLH can find its root in cytokine storm, hyperactivation of tissue-infiltrating macrophage and T cell, as well as acquired defects in cytotoxicity (3, 4). Familial HLH frequently happens within the first year of life and is taken as a genetic disease of impaired perforin­dependent cytotoxic function (5). Meanwhile secondary HLH (sHLH) can present at any age and is usually triggered by infection (i.e., viruses, bacteria, parasites, and fungi), malignancy (hematological malignancy in particular), autoimmune disorders, and hematological and solid organ transplantation (6). Autoimmune-related sHLH, or macrophage activation syndrome (MAS), is most commonly seen in adult-onset Still’s disease (AOSD), systemic juvenile idiopathic arthritis (sJIA), systemic lupus erythematosus (SLE) and Kawasaki disease (KD) (7, 8).

Idiopathic inflammatory myopathy (IIM) is a group of autoimmune disorders characterized by impairment of skin and bilateral proximal skeletal muscle (9, 10). Multiple extracutaneous and extramuscular complications encompassing interstitial lung disease, infection, carcinoma, etc. contributed to low quality of life, increased medical expense, and unfavorable outcome of IIM patients (11). In terms of sHLH in IIM patients, published literatures were mostly case reports or systemic review (12). In our previous work (13), the incidence of sHLH in adult IIM patients was merely 4.2%. However, the short-term mortality rate in IIM patients with sHLH was 77.8%, and missed diagnosis was quite common in clinical practice. With much remains unknown in these patients, it is thus necessary to continuously dig into the rare but fatal complication to figure out biomarkers and tools for early identification as well as impairment beyond bone marrow since IIM and sHLH were both systemic crises.

Recent years have seen cytokines and PET/CT scan as effective tools in prediction and evaluation of sHLH (14, 15). Since ^18^F-fluorodeoxyglucose (FDG) uptake in PET/CT scan can, to some extent, reflect focal inflammation and immune activation, the clinical value of PET/CT scan has been evaluated in familial and secondary HLH patients (16). Published literatures about PET/CT scan in IIM patients, nevertheless, mostly focused on its value in cancer screening, measurement of muscular impairment, and differentiation of myositis phenotypes (17). The clinical value of PET/CT scan in development of sHLH in IIM patients remains unknown. Meanwhile, the clinical value of cytokines like interleukin (IL)-6, IL-10, IL-18, and interferon (IFN)-γ have been revealed in MAS secondary to sJIA or KD (15, 18). In IIM patients with sHLH, however, the role and clinical value of cytokine profiles were still enigmas.

To fill in the gap in the field of IIM and sHLH, we sought to clarify the potential value of PET/CT scan and cytokine profiles in prediction and early identification of sHLH in IIM patients, as well as the distinctive radiological manifestations and the regarding pathophysiological alterations hidden in these patients.

## Patients and Methods

### Patients

To construct a retrospective cohort, we retrieved medical records and follow-up documents of adult patients who were hospitalized at Qingchun, Zhijiang, Yuhang, and Chengzhan divisions of FAHZJU with discharge diagnosis of dermatomyositis (DM), polymyositis (PM), and amyopathic dermatomyositis (ADM) from January 1, 2017 to December 31, 2020. The inclusion criteria of this study were as follows: (1) age over 18 years old; (2) the definite/probable diagnosis of DM, PM, or ADM satisfied the 2017 ACR/EULAR classification criteria, as certified by two experienced rheumatologists (Heng Cao and Bei Xu) (19); and (3) PET/CT scan performed during hospitalization. Exclusion criteria were as follows: (1) confirmed overlap syndromes with other connective tissue diseases (CTDs); (2) myopathy related to strenuous exercise, thyroid dysfunction, drug-induced myositis (i.e., statins, lamivudine, and Chinese herbal medicine), inherited metabolic disorders, etc.; (3) newly identified or unresolved malignancies; (4) hospitalization for reasons unrelated to myositis and its complications such as fracture, pregnancy, acquired immunodeficiency syndrome, and cataract, due to insufficient medical records for this study; and (5) loss to follow-up without death from any cause within 6 months after hospitalization. The research protocol was approved by the Institutional Review Board (IRB) of FAHZJU (Reference Number: 2021-194) and was implemented in accordance with the Declaration of Helsinki. Written informed consent to utilize and publish clinical data was acquired from all the patients involved at hospitalization.

### Clinical Assessments

Clinical records of all the enrolled patients were retrospectively screened and collected utilizing the electronic medical record (EMR) system of FAHZJU. Data including demographic information, disease activity assessment, complications, immunosuppressive regimens, and radiological/laboratory findings were acquired and analyzed. Survival data were extracted from the follow-up records. To be exact, IIM patients were followed from the date of hospitalization until the end of follow-up. For patients who died during hospitalization, their dates of death were accurately documented in the EMR system. For patients who were discharged, a routine return visit was arranged 2 weeks after discharge. In addition to the regular inpatient or outpatient visits, a concise telephone interview was carried out 3 months after discharge, and at an annual frequency afterwards. The end of follow-up could be owing to death from any cause, loss to follow-up, or closure of follow-up for the purpose of this study (June 30, 2021).

Baseline disease activity assessment, lung function testing, and laboratory and radiological detections were performed in the first week of hospitalization. On-admission IIM disease activity was routinely measured using the Myositis Disease Activity Assessment Visual Analogue Scales (MYOACT) (13). ILD and its rapid progression were checked and confirmed by an experienced radiologist and a respiratory specialist (YL and BY) using lung HRCTs. A subset of RP-ILD patients was defined as those presenting with progressive dyspnea and progressive hypoxemia, acute worsening of interstitial alteration on the lung radiograph within 1 month after hospitalization, or onset of respiratory symptoms (20–22). Diagnosis of sHLH was made according to the criteria proposed by the Histiocyte Society in 2004 (HLH-2004, **Supplementary Table 1**) (13, 23) and was confirmed by a hematologist (BW). The included IIM patients were hereby divided into an HLH group and a non-HLH group (control group). Since pulmonary infection was easily confused with interstitial lung disease, identification of bacterial, fungal, or tuberculosis infection was a careful and comprehensive decision based on the essential microbiological findings in sputum or blood, clinical manifestations, and radiographic and laboratory abnormalities. Besides, diagnosis of Epstein–Barr virus (EBV) and cytomegalovirus (CMV) infection relied on the detection of serum antibody and DNA. All of the included patients received potent immunosuppressive therapies: (1) systemic prednisolone (PSL) or methylprednisolone (mPSL) with a maximum dosage ≥ 1 mg/kg/day (calculated by prednisolone); (2) combined therapy of PSL/mPSL, disease-modifying anti-rheumatic drugs (DMARDs), or Janus kinase (JAK) inhibitors, with or without intravenous immunoglobulin (IVIG). The DMARDs used in these patients encompassed Mycophenolate, Tacrolimus, Cyclosporine, Methotrexate, Cyclophosphamide, Thalidomide, and Hydroxychloroquine. Meanwhile the JAK inhibitors used in these patients included Tofacitinib and Baricitinib.

### Laboratory Detections

To acquire the profiles of myositis-specific antibodies (MSAs) and myositis-associated antibodies (MAAs) as well as peripheral lymphocyte subsets and cytokines, serum samples were routinely acquired and detected within the first week of hospitalization. The 12 MSAs (anti-MDA5, anti-TIF1γ, anti-Jo-1, anti-EJ, anti-OJ, anti-PL-7, anti-PL-12, anti-Mi-2α, anti-Mi-2β, anti-SAE1, anti-SRP, and anti-NXP2) and 4 MAAs (anti-Ro-52, anti-PM-Scl75, anti-PM-Scl100, anti-Ku) were assessed by an immunoblotting assay utilizing the EUROLINE Autoimmune Inflammatory Myopathies 16 Ag (IgG) commercial line blot assay (Euroimmun, Lübeck, Germany) including a membrane strip with the 16 autoantigens as per the manufacturer’s instructions. Peripheral lymphocyte subsets and cytokine profiles were detected by the Department of Clinical Laboratory (FAHZJU) and uploaded onto the EMR system (detected values). Specifically, peripheral lymphocyte subsets were determined as percentages of CD3^+^CD4^+^, CD3^+^CD8^+^, CD3^-^CD16^+^CD56^+^, and CD3^-^ CD19^+^ cells using CD45-PE, CD3-PC5, CD4-FITC, CD8-PE, CD3-FITC-CD(16 + 56)-PE, and CD19-FITC mouse anti-human fluorescence monoclonal antibodies (BD Bioscience) and the BD FACScanto™ II flow cytometer (Becton Dickinson, San Jose, CA, USA). Meanwhile serum levels of IL-2, IL-4, IL-6, IL-10, IL-17A, tumor necrosis factor (TNF)-α, and IFN-γ were quantified utilizing the cytometric bead array (CBA) kit BD™ CBA Human Th1/Th2/TH17 Cytokine Kit (BD Biosciences, San Jose, CA, USA) and the flow cytometer (described above). Measurement of cytokine was implemented as per the manufacturer’s instructions. The lower and upper limits of cytokine detection were 0.10 pg/ml and 5,000.00 pg/ml, respectively. The data were engendered in graphical and tabular format utilizing FCAP Array™ software (BD Biosciences, San Jose, CA, USA).

### PET/CT Scan

Whole-body detection of CT and PET, which was performed with a combined PET/CT scanner (Biograph, Sensation 16, Siemens systems), covered a wide range from the meatus of the ear to the mid-thigh. Patients fasted overnight or for at least 6 h prior to the PET/CT detection. Blood glucose levels were confirmed to be within normal limits before the injection of 4.0 MBq/kg of [18F] FDG. Patients rested for 30 min so that non-specific FDG uptake in muscles was minimized. Imaging acquisition was systematically performed at 60 min after injection. SUV (standard uptake value) was calculated by the following formula: SUV (g/ml) = regional radioactivity concentration (Bq/ml)/[injected dose (Bq)/body weight (g)]. Region of interest (ROI, 20 mm diameter) was manually placed by a single trained radiologist (YL) at the region with the highest FDG uptake in the following organs: liver, spleen, bone marrow (thoracic, T10–T12, lumbar, L2–L4) (24), bilateral lung, esophagus, stomach, small intestine, colon/rectum, bilateral cerebellum, and bilateral proximal muscles (namely, trapezius, deltoid, biceps, iliopsoas, gluteus medius, gluteus maximus, and quadriceps) (25), excluding the region markedly influenced by FDG uptake in adjacent anatomical structures. In order to avoid noise and acquire the value representing a certain volume of targeted organs, SUV was calculated as the mean value of ROI (SUVmean) rather than the maximum value at a single pixel (25). For bilaterally distributed organs, the SUVmean was counted as the maximum SUVmean value of the symmetrical sides. The radiologist was blinded to the diagnostic subtypes, complications, and outcome of the included patients when assessing the SUVmean value of each and every targeted organ.

### Statistical Analysis

Statistical analysis was performed using SPSS 22.0 (Chicago, IL, USA), R 3.6.1, and GraphPad Prism 8.0. In comparison between HLH and non-HLH patients, independent sample *t*-test was utilized to compare normally distributed continuous variables; meanwhile Mann–Whitney *U* test was used to compare skewed continuous variables or ordinal categorical variables. Chi-square test and Fisher’s exact test were utilized to compare unordered categorical variables. *p*-values in comparisons and univariate analyses were adjusted by false discovery rate (FDR) correction, using p.adjust function in R.3.6.1, to acquire adjusted *p*-values and minimize type I error. The univariate and the following multivariate logistic regression analyses were used to identify significant factors associated with development of sHLH in IIM patients. Factors with *p* < 0.05 in univariate analyses or comparisons were entered into the multivariate analysis. Binary logistic regression was also used to evaluate the contributary roles of statistically significant factors to development of sHLH. Receiver operating characteristic (ROC) curve analysis was applied to quantify the predictive value of continuous variables. The correlation between two continuous variables was measured by the Pearson linear analysis. Survival in different groups was evaluated by the Kaplan–Meier method with log-rank test. All tests were two-sided, and *p* <  0.05 was considered statistically significant.

## Results

### Patient Characteristics

**Figure 1:**
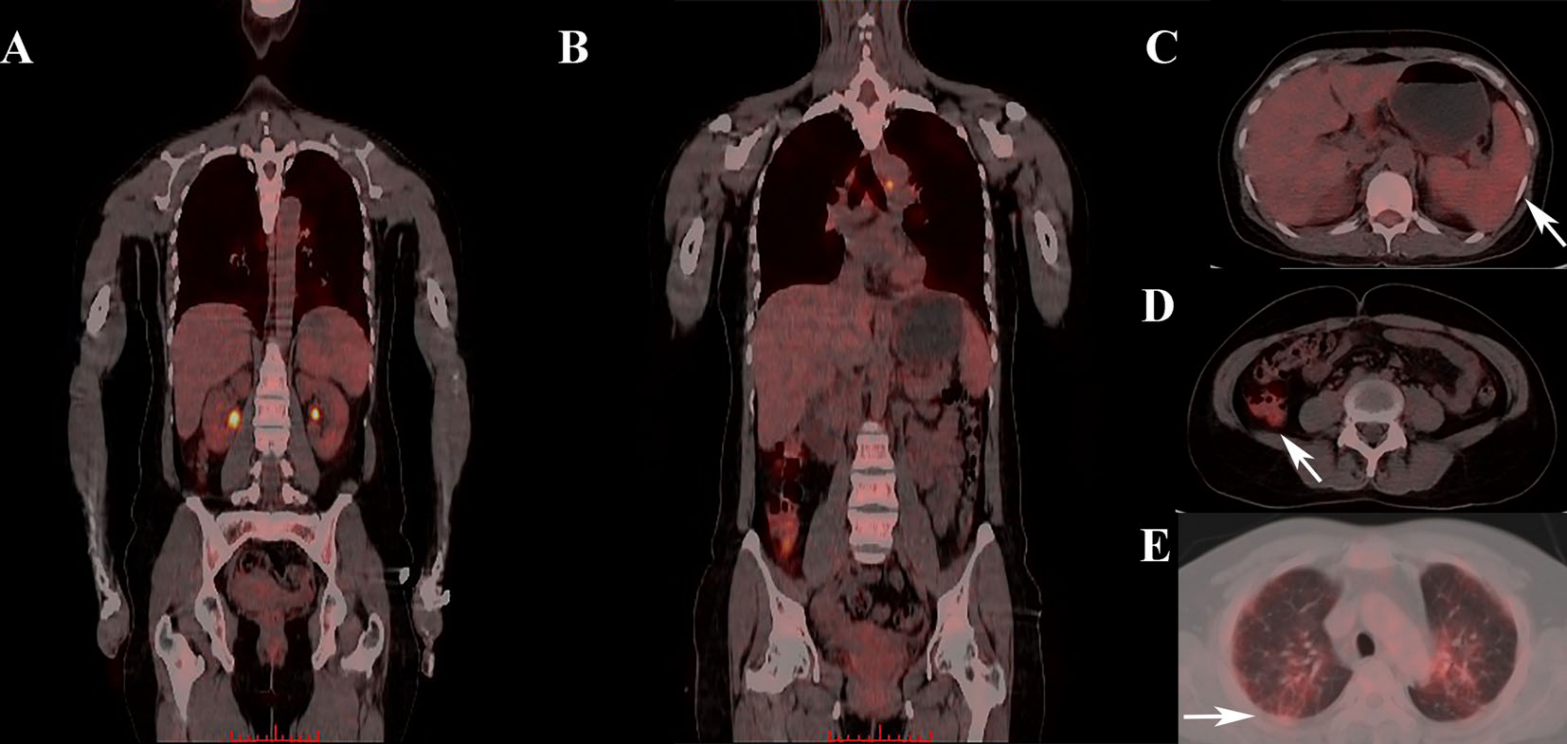
Abnormal FDG uptake in one IIM patient who was later diagnosed with sHLH. **(A, B)** Whole-body PET/CT scan showing abnormal FDG uptake in multiple organs. **(C)** Elevated splenic FDG uptake in PET/CT scan (where the arrow pointed). **(D)** Elevated colon FDG uptake in PET/CT scan (where the arrow pointed). **(E)** Elevated pulmonary FDG uptake in PET/CT scan (where the arrow pointed). FDG, Fluorodeoxyglucose; IIM,diopathic inflammatory myopathy; sHLH, Secondary hemophagocytic lymphohistiocytosis.

**Figure 2:**
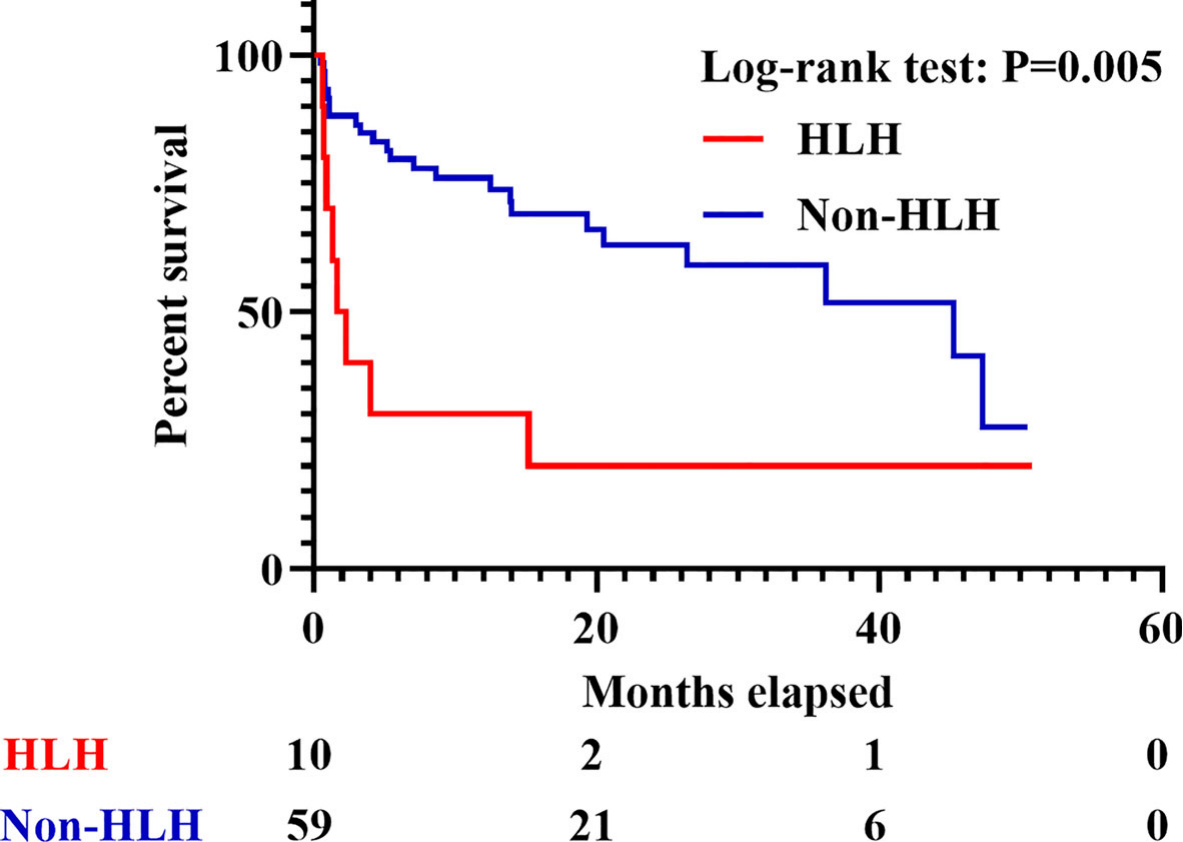
Survival of IIM patients with or without sHLH. IIM, Idiopathic inflammatory myopathy; sHLH, Secondary hemophagocytic lymphohistiocytosis.

A total of 352 adult IIM patients, including 11 patients who developed sHLH (3.1%), were hospitalized at the Qingchun, Zhijiang, Yuhang, and Chengzhan divisions of FAHZJU from January 1, 2017 to December 31, 2020. Among them, 69 patients who satisfied the inclusion/exclusion criteria were finally included into the study (**Supplementary Figure 1**), encompassing 45 with DM, 12 with PM, and 12 with ADM. Twenty-eight (40.6%) were males, and the mean age of all the patients included was 56.52 ± 11.71 years old. Thirty-one patients (44.9%) died in follow-up and the medium follow-up time was 13.33 (4.70, 26.45) months. Among the 69 patients, 10 patients developed sHLH (**Figure 1** and **Supplementary Tables 2** and **3**), and the 59 patients without sHLH constituted the control group. Eight of the 10 sHLH patients were anti-MDA5 antibody positive, while the remaining two patients were both complicated with bacterial infection beforehand and were anti-PL-7 antibody positive and anti-SRP antibody positive, respectively. Besides, all of the sHLH events were identified after performance of PET/CT scan. IIM patients complicated with sHLH were found to suffer from worse survival (*p* = 0.005, **Figure 2**) with seven patients (70.0%) perished within 6 months. In patients who developed sHLH, infection (50.0%) was the most common contributor to death. Meanwhile exacerbation of ILD (including RP-ILD, 39.1%) was identified as the most frequent cause for death in the non-HLH group, followed by carcinoma (21.7%) (**Supplementary Table 4**). Three of the IIM patients with sHLH received etoposide therapy; however, none of them survived beyond 3 months (**Supplementary Table 5**). Apart from the conventional therapy of steroid, DMARDs, and IVIG, four patients received combined therapy of steroid and JAK inhibitors (two with Tofacitinib and two with Baricitinib). None of the four patients under the medication of steroid and JAK inhibitors developed sHLH.

### Clinical Factors Correlated with sHLH

**Figure 3:**
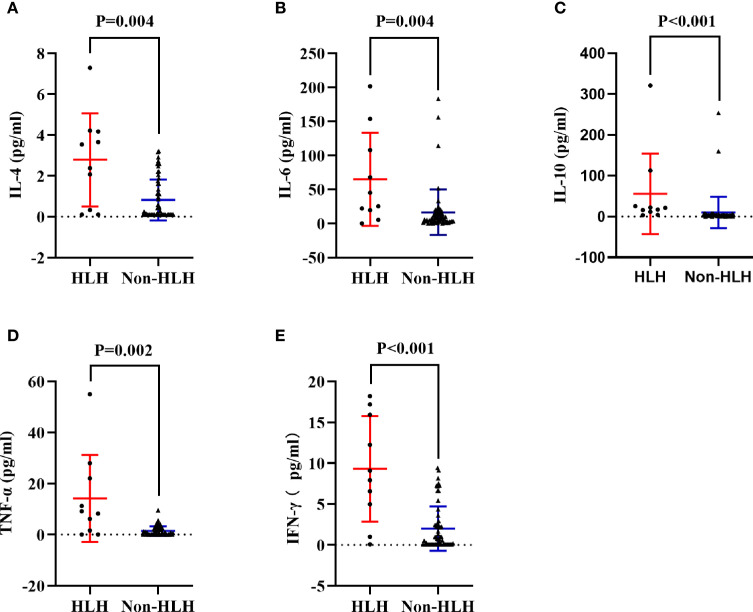
Comparisons of cytokines in HLH and non-HLH groups. **(A)** Comparison of serum IL-4 between HLH and non-HLH groups. **(B)** Comparison of serum IL-6 between HLH and non-HLH groups. **(C)** Comparison of serum IL-10 between HLH and non-HLH groups. **(D)** Comparison of serum TNF-α between HLH and non-HLH groups. **(E)** Comparison of serum IFN-γ between HLH and non-HLH groups. HLH, Hemophagocytic lymphohistiocytosis; IL, Interleukin; TNF,Tumor necrosis factor; IFN, Interferon.

**Figure 4:**
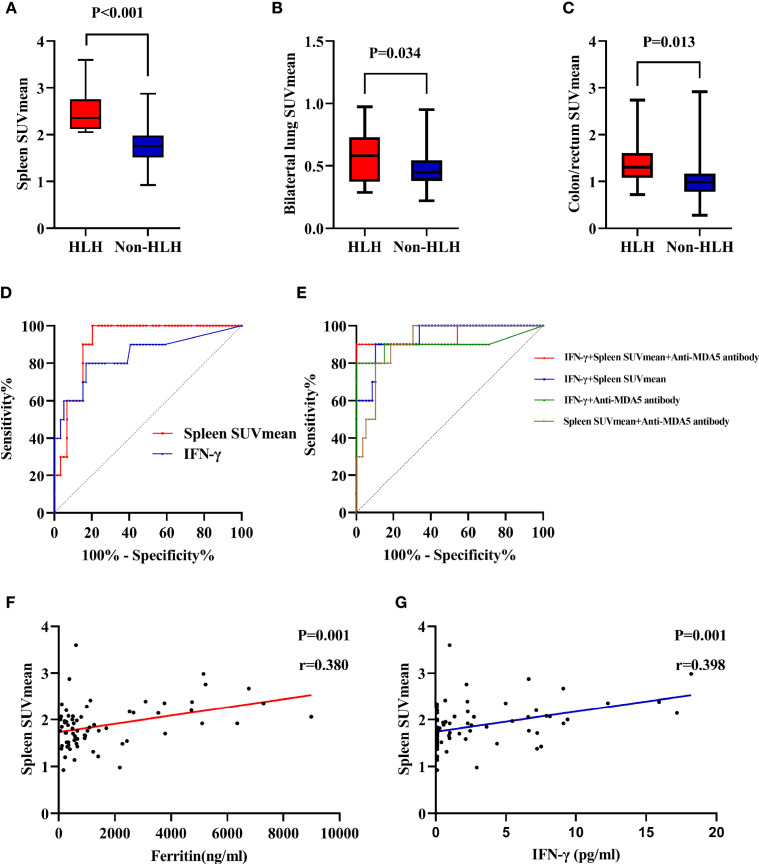
Evaluation of ^18^F-FDG uptake in IIM patients with or without sHLH. **(A)** Comparison of spleen SUVmean between HLH and non-HLH groups. **(B)** Comparison of bilateral lung SUVmean between HLH and non-HLH groups. **(C)** Comparison of colon/rectum SUVmean between HLH and non-HLH groups. **(D)** ROC curve analysis of spleen SUVmean and IFN-γ in predicting sHLH. **(E)** ROC curve analysis of combination of two or three statistically significant parameters in predicting sHLH. **(F)** Correlation of spleen SUVmean and serum ferritin. **(G)** Correlation of spleen SUVmean and serum IFN-γ. ^18^F-FDG, ^18^F-fluorodeoxyglucose; IIM, Idiopathic inflammatory myopathy; sHLH, Secondary hemophagocytic lymphohistiocytosis; SUVmean, mean standard uptake value; ROC, Receiver operating characteristic; IFN, Interferon.

To acquire an initial understanding on clinical factors related to sHLH in IIM patients, a comparison between IIM patients with or without sHLH was carried out and identified that patients who developed sHLH had a female dominance (*p* = 0.041); more complication of pulmonary bacterial infection (*p* = 0.011); higher disease activity (*p* = 0.016); lower percentage of peripheral CD3^-^CD16^+^CD56^+^ lymphocytes (*p* = 0.024); higher serum levels of IL-4 (*p* = 0.004, **Figure 3A**), IL-6 (*p* = 0.004, **Figure 3B**), IL-10 (*p* < 0.001, **Figure 3C**), TNF-α (*p* = 0.002, **Figure 3D**), and IFN-γ (*p* < 0.001, **Figure 3E**); higher serum levels of alanine transaminase (ALT, *p* = 0.023) and aspartate transaminase (AST, *p* = 0.010); higher levels of spleen SUVmean (*p* < 0.001, **Figure 4A**), bilateral lung SUVmean (*p* = 0.034, **Figure 4B**), colon and rectum SUVmean (*p* = 0.013, **Figure 4C**); as well as positivity of anti-MDA5 antibody (*p* = 0.005). After FDR correction, nevertheless, only a few remained statistically significant, including higher spleen SUVmean (*p* < 0.001) and positivity of anti-MDA5 antibody (*p* = 0.049), as well as higher serum levels of IL-4 (*p* = 0.045), IL-6 (*p* = 0.045), IL-10 (*p* < 0.001), TNF-α (*p* = 0.034), and IFN-γ (*p* < 0.001) (**Table 1**).

**Table 1:** Comparisons of multiple factors between HLH and non-HLH groups.

Factors	HLH (10)	Non-HLH (59)	*p*-value	*p*-adjusted
**Age (y)**	55.90 ± 9.39	56.63 ± 12.12	0.857	1.000
**Sex (male/female)**	1/9	27/32	0.041	0.186
**Clinical manifestations or complications**				
**Pulmonary bacterial infection**	5 (50.0%)	7 (11.9%)	0.011	0.083
**Pulmonary fungal infection**	2 (20.0%)	7 (11.9%)	0.674	0.977
**Tuberculosis infection**	0 (0.0%)	1 (1.7%)	1.000	1.000
**EBV infection**	1 (10.0%)	13 (22.0%)	0.653	0.977
**CMV infection**	0 (0.0%)	3 (5.1%)	1.000	1.000
**RP-ILD**	4 (40.0%)	17 (28.8%)	0.479	0.958
**Carcinoma**	0 (0.0%)	11 (18.6%)	0.345	0.916
**Disease activity**				
**MYOACT score**	14.50 (8.75, 16.25)	9.00 (7.00, 12.00)	0.016	0.099
**Laboratory findings**				
**CD3^+^CD4^+^ lymphocytes (%)**	36.61 ± 13.80	40.55 ± 13.47	0.397	0.916
**CD3^+^CD8^+^ lymphocytes (%)**	28.75 (19.88, 39.60)	20.50 (15.60, 30.10)	0.067	0.268
**CD4^+^/CD8^+^ Ratio**	1.35 (0.68, 2.14)	1.73 (1.29, 3.05)	0.147	0.476
**CD3^-^CD16^+^CD56^+^ lymphocytes (%)**	7.90 (1.88, 9.15)	11.00 (6.60, 20.00)	0.024	0.126
**CD3^-^CD19^+^ lymphocytes(%)**	21.42 ± 11.73	19.73 ± 11.76	0.675	0.977
**IL-2 (pg/ml)**	0.68 (0.10, 7.64)	0.22 (0.10, 1.80)	0.473	0.958
**IL-4 (pg/ml)**	2.96 (0.27, 4.18)	0.15 (0.10, 1.60)	0.004	0.045
**IL-6 (pg/ml)**	35.25 (16.10, 119.41)	6.62 (2.84, 14.66)	0.004	0.045
**IL-10 (pg/ml)**	19.32 (10.14, 47.29)	3.28 (1.72, 4.25)	<0.001	<0.001
**TNF-α (pg/ml)**	8.69 (1.26, 23.53)	0.65 (0.10, 2.23)	0.002	0.034
**IFN-γ (pg/ml)**	8.51 (4.00, 16.24)	0.70 (0.10, 2.54)	<0.001	<0.001
**IL-17A (pg/ml)**	2.72 (0.10, 15.30)	0.10 (0.10, 4.03)	0.208	0.643
**CRP (mg/L)**	3.70 (1.14, 7.66)	6.70 (3.10, 33.00)	0.102	0.383
**ESR (mm/h)**	28.00 (8.00, 62.50)	16.00 (9.00, 30.00)	0.418	0.917
**ALT (U/L)**	134.00 (76.25, 335.00)	62.00 (25.00, 114.00)	0.023	0.126
**AST (U/L)**	190.50 (82.25, 908.00)	51.00 (32.00, 132.00)	0.010	0.083
**LDH (U/L)**	371.00 (290.25, 566.50)	329.00 (250.00, 508.00)	0.404	0.916
**CK (U/L)**	101.50 (48.00, 526.50)	106.00 (52.00, 457.00)	0.946	1.000
**^18^F-FDG PET/CT scan findings**				
**Bilateral lung SUVmean**	0.58 ± 0.21	0.47 ± 0.14	0.034	0.165
**Liver SUVmean**	1.59 ± 0.31	1.73 ± 0.46	0.367	0.916
**Spleen SUVmean**	2.48 ± 0.49	1.77 ± 0.39	<0.001	<0.001
**Bone marrow SUVmean**	1.87 ± 0.63	1.63 ± 0.42	0.128	0.435
**Cardiac SUVmean**	1.25 (1.07, 1.70)	1.58 (1.07, 2.60)	0.310	0.916
**Esophagus SUVmean**	1.24 (1.07, 1.64)	1.39 (1.09, 1.77)	0.664	0.977
**Stomach SUVmean**	0.79 (0.51, 1.30)	0.99 (0.78, 1.17)	0.331	0.916
**Small intestine SUVmean**	1.06 ± 0.23	1.03 ± 0.33	0.798	1.000
**Colon and rectum SUVmean**	1.31 (1.08, 1.61)	0.99 (0.78, 1.07)	0.013	0.088
**Bilateral cerebellum SUVmean**	5.19 ± 1.74	5.15 ± 1.52	0.943	1.000
**Bilateral trapezius SUVmean**	0.70 (0.64, 0.85)	0.84 (0.71, 1.07)	0.107	0.383
**Bilateral deltoid SUVmean**	0.75 (0.65, 0.81)	0.79 (0.61, 1.10)	0.523	0.958
**Bilateral biceps SUVmean**	0.79 (0.66, 1.00)	0.79 (0.65, 1.15)	0.621	0.977
**Bilateral ilioposas SUVmean**	1.23 ± 0.37	1.15 ± 0.38	0.529	0.958
**Bilateral gluteus maximus SUVmean**	0.79 (0.61, 1.06)	0.76 (0.57, 0.96)	0.506	0.958
**Bilateral gluteus medius SUVmean**	0.87 (0.75, 0.97)	0.94 (0.73, 1.21)	0.404	0.916
**Bilateral quadriceps SUVmean**	0.78 (0.71, 1.02)	0.82 (0.69, 1.04)	0.772	1.000
**Myositis-specific antibodies and myositis-associated antibodies**				
**Anti-MDA5**	8 (80.0%)	18 (30.5%)	0.005	0.049
**Anti-PL-7**	1 (10.0%)	6 (10.2%)	1.000	1.000
**Anti-PL-12**	0 (0.0%)	2 (3.4%)	1.000	1.000
**Anti-EJ**	0 (0.0%)	2 (3.4%)	1.000	1.000
**Anti-OJ**	0 (0.0%)	1 (1.7%)	1.000	1.000
**Anti-Jo-1**	0 (0.0%)	7 (11.9%)	0.582	0.958
**Anti-TIF1γ**	0 (0.0%)	6 (10.2%)	0.582	0.958
**Anti-Mi-2α**	0 (0.0%)	2 (3.4%)	1.000	1.000
**Anti-Mi-2β**	0 (0.0%)	4 (6.8%)	1.000	1.000
**Anti-SAE1**	0 (0.0%)	6 (10.2%)	0.582	0.958
**Anti-NXP2**	0 (0.0%)	8 (13.6%)	0.592	0.958
**Anti-SRP**	1 (10.0%)	2 (3.4%)	0.380	0.916
**Anti-Ku**	0 (0.0%)	2 (3.4%)	1.000	1.000
**Anti-PM-Scl75**	0 (0.0%)	4 (6.8%)	1.000	1.000
**Anti-PM-Scl100**	0 (0.0%)	0 (0.0%)	NA	NA
**Anti-Ro-52**	6 (60.0%)	27 (45.8%)	0.502	0.958
**Therapies**				
**Steroid monotherapy**	3 (30.0%)	20 (33.9%)	1.000	1.000
**Steroid+DMARDs**	4 (40.0%)	24 (40.7%)	1.000	1.000
**Steroid+IVIG**	3 (30.0%)	4 (6.8%)	0.057	0.242
**Steroid+DMARDs+IVIG**	0 (0.0%)	7 (11.9%)	0.582	0.958
**Steroid+JAK inhibitor**	0 (0.0%)	4 (6.8%)	1.000	1.000
**IIM subtypes**				
**DM**	6 (60.0%)	39 (66.1%)	0.730	1.000
**PM**	2 (20.0%)	10 (16.9%)	1.000	1.000
**ADM**	2 (20.0%)	10 (16.9%)	1.000	1.000

HLH, Hemophagocytic lymphohistiocytosis; p-adjusted, Adjusted p-value after false discovery rate correction; y, years; EBV, Epstein–Barr virus; CMV, Cytomegalovirus; RP-ILD, Rapidly progressive interstitial lung disease; MYOACT, Myositis Disease Activity Assessment Visual Analogue Scales; CD, Clusters of differentiation; IL, Interleukin; TNF, Tumor necrosis factor; IFN, Interferon; CRP, C-reactive protein; ESR, Erythrocyte sedimentation rate; ALT, Alanine transaminase; AST, Aspartate transaminase; LDH, Lactate dehydrogenase; CK, Creatine kinase; FDG, Fluorodeoxyglucose; SUVmean, mean standard uptake value; NA, Not available; DMARDs, Disease-modifying anti-rheumatic drugs; IVIG, Intravenous immunoglobulin; JAK, Janus kinase; IIM, Idiopathic inflammatory myopathy; DM, dermatomyositis; PM, Polymyositis; ADM, Amyopathic dermatomyositis.

The univariate logistic regression analyses for sHLH revealed that pulmonary bacterial infection (*p* = 0.007), disease activity (*p* = 0.012), percentage of peripheral CD3^-^CD16^+^CD56^+^ lymphocytes (*p* = 0.040), IL-4 (*p* = 0.002), IL-6 (*p* = 0.007), IL-10 (*p* = 0.049), TNF-α (*p* = 0.003), IFN-γ (*p* = 0.001), AST (*p* = 0.011), spleen SUVmean (*p* = 0.001), bilateral lung SUVmean (*p* = 0.045), colon/rectum SUVmean (*p* = 0.042), anti-MDA5 antibody (*p* = 0.008), and use of PSL/mPSL+IVIG (*p* = 0.040) were associated with development of sHLH in IIM patients (**Supplementary Table 6**). The following multivariate logistic regression analysis identified IFN-γ (*p* = 0.017), spleen SUVmean (*p* = 0.035), and anti-MDA5 antibody (*p* = 0.049) as clinical factors that were significantly correlated with development of sHLH in IIM patients (**Supplementary Table 7**).

### Predictors for sHLH

Utilizing ROC curve analyses, the optimal cutoff value of IFN-γ for sHLH was >4.68 pg/ml, with a sensitivity of 80.0%, a specificity of 83.1%, and an AUC of 0.840 (**Figure 4D**). Meanwhile the optimal cutoff value of spleen SUVmean for sHLH was >2.029, with a sensitivity of 100.0%, a specificity of 79.7%, and an AUC of 0.910 (**Figure 4D**). To establish a more efficient and balanced predictor for development of sHLH, a series of models were constructed using logistic regression analysis about IFN-γ, spleen SUVmean, and anti-MDA5 antibody (two or three factors). In the present study, the coefficients were as follows: −10.274 + 0.343 * IFN-γ + 3.226 * spleen SUVmean for IFN-γ and spleen SUVmean (optimal cutoff value of −1.586, sensitivity of 90.0%, specificity of 89.8%, and AUC of 0.937); −5.890 + 0.511 * IFN-γ + 3.222 * anti-MDA5 antibody for IFN-γ and anti-MDA5 antibody (optimal cutoff value of −0.468, sensitivity of 80.0%, specificity of 100.0%, and AUC of 0.899); −11.182 + 3.952 * spleen SUVmean + 1.982 * anti-MDA5 antibody for spleen SUVmean and anti-MDA5 antibody (optimal cutoff value of −1.888, sensitivity of 90.0%, specificity of 81.4%, and AUC of 0.912); −12.453 + 0.492 * IFN-γ + 3.154 * spleen SUVmean and 2.943 * anti-MDA5 antibody for combination of the three factors (optimal cutoff value of 0.047, sensitivity of 90.0%, specificity of 100.0%, and AUC of 0.946) (**Figure 4E**). Through Pearson linear analysis, a moderate correlation was identified between ferritin and spleen SUVmean (*p* = 0.001, *r* = 0.380, **Figure 4F**) as well as IFN-γand spleen SUVmean (*p* = 0.001, *r* = 0.398, **Figure 4G**).

### Identification of Survival-Related Factors

**Figure 5:**
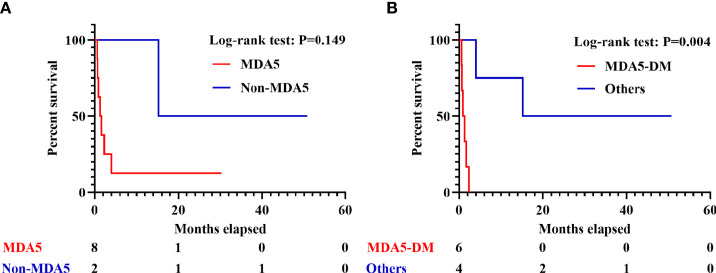
Survival analyses in IIM patients with sHLH. **(A)** Survival of sHLH patients with or without positivity of anti-MDA5 antibody. **(B)** Survival of anti-MDA5-positive DM patients and the other patients. IIM, Idiopathic inflammatory myopathy; sHLH, Secondary hemophagocytic lymphohistiocytosis; DM, dermatomyositis.

To probe into the predictive value of PET/CT scan in survival, the 10 IIM patients with sHLH were further divided into two groups depending on whether he or she died within 3 months (six patients) or survived beyond this threshold (four patients). Only age (*p* = 0.019), higher level of serum creatine kinase (CK, *p* = 0.038), and DM subtype (*p* = 0.005) exhibited statistical significance. However, the significance vanished after FDR correction (**Supplementary Table 8**). In the six patients who perished within 3 months, all of them were anti-MDA5-antibody-positive DM patients. Due to the limitation of sample size, we failed to identify significant difference in anti-MDA5-antibody-positive/negative IIM patients who developed sHLH (*p* = 0.149, **Figure 5A**). By narrowing down to anti-MDA5-antibody-positive DM patients (*vs*. other patients), a significant difference was identified in survival (*p* = 0.004, **Figure 5B**).

## Discussion

To date, this is the first cohort study to systemically investigate the clinical value of whole-body PET/CT scan in sHLH among adult IIM patients. Based on data from four centers, the incidence of sHLH was 3.1% in adult IIM patients, which was similar to the preceding finding (4.2%) but much lower compared with those in AOSD (approximately 15.0%) and sJIA (10.0%–30.0%) (7, 13). Rare as it is, the 6-month mortality of IIM patients with sHLH was 70.0%, indicating a higher mortality than those in other rheumatic diseases (7, 12).

Preceding studies on PET/CT scan of acute-myelocytic-leukemia-related HLH patients revealed elevated FDG uptakes in spleen and bone marrow (14). In IIM patients who developed sHLH, only spleen SUVmean was prominently elevated and significantly associated with development of sHLH. The hemophagocytosis by activated macrophages occurs in both bone marrows and spleen (26). Increased splenic FDG uptake could be taken as a manifestation of immune cell (macrophage and T cell included) activation in sHLH (27). Meanwhile, the moderate correlation between splenic FDG uptake and serum ferritin level as well as serum IFN-γconstituted a further demonstration for immune activation. The role of aggravated splenic immune activation in development of sHLH in IIM patients was hereby revealed. In contrast, the elevated bone marrow FDG uptake could contribute to the active proliferation of hematopoietic cells (28). The different disease background and different causes for altered FDG uptake in spleen and bone marrow might explain the insignificant difference of bone marrow SUVmean between the two groups of IIM patients. Additionally, in the IIM cohort, spleen SUVmean was found to work as a more satisfying predictor for sHLH than serum IFN-γlevel with larger AUC and better sensitivity. A more efficient and balanced predicting tool was initially developed when combining serum IFN-γ, spleen SUVmean, and anti-MDA5 antibody. Despite the cost and the radiation exposure of PET/CT scan, the clinical value of splenic FDG uptake deserved further exploration since sHLH was frequently omitted in IIM patients. Nevertheless, splenic FDG uptake was not found effective in predicting outcome of IIM patients with sHLH.

Apart from the elevation of spleen SUVmean, increased FDG uptakes of lung and colon/rectum were also recognized in IIM patients with sHLH before FDR correction. It is known that sHLH is a systemic inflammatory disorder featuring macrophage and T-cell activation, cytokine storm, as well as a consequent hemophagocytosis (29). Extra-hematological involvements have thus drawn attention from physicians and researchers. Frequent interstitial infiltrates (54.0%) have already been identified in a large sHLH cohort, and was found to contribute to MAS development by excessively amplifying the pathogenic proinflammatory response (30, 31). The significantly elevated pulmonary FDG uptake, which indicated focal immune activation, might work as an additional demonstration for aggravated pulmonary involvement in IIM patients with sHLH. In addition to sHLH and autoimmune-related ILD, activation of T cells and macrophages was also identified to play a key pathogenic role in the development of inflammatory bowel disease (IBD) (32–34). Elevated colon/rectum FDG uptake might therefore suggest exacerbated gastrointestinal involvement in IIM patients with sHLH.

Abundant secretion of various cytokines has been recognized as a typical manifestation in HLH patients. Serum IFN-γ, TNF-α, IL-4, IL-6, IL-10, and IL-18 were found to be valuable in early identification, disease progression, and outcome prediction of HLH, especially in pediatric patients, EBV-related HLH, and MAS associated with sJIA or KD (8, 15, 18, 35–38). IFN-γ, in particular, was identified as a major macrophage-activating signal that would initiate a robust release of cytokines and chemokines (8). Meanwhile therapeutic regimen targeting IFN-γ has been testified effective in optimal control of HLH (39). In this study, serum levels of IL-4, IL-6, IL-10, TNF-α, as well as IFN-γ were observed elevated in IIM patients who were later diagnosed with sHLH. However, we cannot ignore that IIM patients had a profound immunological abnormality background and were frequently complicated with infection, ILD, and RP-ILD (40–43). Serum cytokines included in this study could also be affected by IIM itself as well as its complications. After adjusting for disease activity, infection, and RP-ILD, only IFN-γ was found significantly correlated with development of sHLH in IIM patients, which was consistent with preceding findings in sJIA (8, 15, 38, 44). However, the clinical value of IFN-γ in predicting sHLH in IIM patients was inferior to spleen SUVmean and demanded further verification in the future.

Anti-MDA5 antibody was comparably more often seen in Asian DM or ADM patients (45, 46). The positivity of anti-MDA5 antibody heralds complication of early and rapidly progressive ILD, and usually leads to unfavorable outcome (47). Its association with sHLH, however, has never been elaborated. In this study, the positivity of anti-MDA5 antibody was found significantly correlated with sHLH after FDR correction as well as multivariate analysis. This is the first clinical study that directly linked anti-MDA5 antibody with sHLH in IIM patients. RP-ILD, which is frequent in patients with positivity of anti-MDA5 antibody, have been found to be correlated with macrophage activation in IIM-ILD patients (48, 49). It is thus not surprising that recent studies initially unveiled the role of macrophage activation in anti-MDA5-antibody-positive DM or ADM patients, through miRNA–mRNA association analysis and detection of macrophage activation biomarkers (50, 51). The underlying pathophysiology of macrophage activation might explain the association between anti-MDA5 antibody and development of sHLH. Besides, anti-MDA5-antibody-positive patients with sHLH also tended to have unfavorable outcome. Due to limited sample size, however, statistical significance only arose when narrowing down to anti-MDA5-antibody-positive DM patients (*vs*. the others).

Most IIM patients with sHLH were refractory to conventional treatment like steroid, DMARDs, IVIG, plasma exchange, and etoposide. The administration of rituximab (RTX) might result in rapid clinical relief and steroid reduction (12). Recent years have seen several reports on the satisfying response to the JAK inhibitor Ruxolitinib in HLH patients, which might indicate the potent effect of JAK inhibitor on suppressing cytokine cascade (1, 4). In our cohort, four patients received a combined therapy of steroid and JAK inhibitor (Tofacitinib or Baricitinib); none of them developed sHLH in follow-up. The role and mechanism of JAK inhibitor in preventing and curing sHLH in CTD patients demand further exploration.

The retrospective nature, the small sample size, and the selection bias (more severe patients) constituted the major limitations of this study. Generally speaking, patients with higher disease activity, dyspnea, complication of fever, and signs of cytopenia tended to receive whole-body PET/CT scan to exclude malignancy or relapse of malignancy. Lack of detection of soluble CD25 and natural killer cell activity in most cases might lead to underestimation of sHLH in IIM patients. The retrospective nature made it impossible to assess more cytokines like IL-18 and soluble TNF receptor. The scarcity of healthy people receiving PET/CT scan made it impossible to construct a healthy control group. Last but not least, we failed to provide valid suggestions for prevention and treatment of sHLH in IIM patients owing to the serious heterogeneity of therapeutic regimens as well as the rarity of sHLH in this cohort.

## Conclusion

sHLH is a fatal complication in adult IIM patients. Increased serum level of IFN-γ, elevated splenic FDG uptake, and positivity of anti-MDA5 antibody were found to be significantly correlated with development of sHLH in IIM patients. Lung and lower digestive tract might also be affected due to systemic immune activation in these patients. In addition, the combination of splenic FDG uptake, serum IFN-γ, and anti-MDA5 antibody was found valuable in predicting development of sHLH in IIM patients. Among IIM patients with sHLH, anti-MDA5-antibody-positive DM patients showed higher tendency for unfavorable outcome.

## Data Availability Statement

The original contributions presented in the study are included in the article/**Supplementary Material**. Further inquiries can be directed to the corresponding author.

## Ethics Statement

The studies involving human participants were reviewed and approved by The Institutional Review Board of the First Affiliated Hospital, Zhejiang University School of Medicine. The patients/participants provided their written informed consent to participate in this study.

## Author Contributions

JYL contributed to the study design, data collection, statistical analysis, writing, and proofreading of this work. HC contributed to the study design, verification of IIM diagnosis, and proofreading of this study. BW contributed to verification of sHLH and data collection. YL contributed to reevaluation of FDG uptake in multiple organs as well as identification of ILD and RP-ILD. BX contributed to verification of IIM diagnosis. YH and YS contributed to data collection, while BY contributed to identification of ILD and RP-ILD. JL contributed to the study design and proofreading of this study. All authors contributed to the article and approved the submitted version.

## Funding

This study was supported by grants from the National Natural Science Foundation of China (81701602).

## Conflict of Interest

The authors declare that the research was conducted in the absence of any commercial or financial relationships that could be construed as a potential conflict of interest.

## Publisher’s Note

All claims expressed in this article are solely those of the authors and do not necessarily represent those of their affiliated organizations, or those of the publisher, the editors and the reviewers. Any product that may be evaluated in this article, or claim that may be made by its manufacturer, is not guaranteed or endorsed by the publisher.
