# Supplementary material for: Clinical Value of 18F-FDG PET/CT Scan and Cytokine Profiles in Secondary Hemophagocytic Lymphohistiocytosis in Idiopathic Inflammatory Myopathy Patients: A Pilot Study
Source: Front Immunol. 2021 Nov 18;12:745211. doi: 10.3389/fimmu.2021.745211 (PMC8636988; doi:10.3389/fimmu.2021.745211)
Supplement: Supplementary file 1 [file Table_1.docx]

**Supplementary table 1 Revised diagnostic guidelines for HLH [13]**

HLH: Haemophagocytic lymphohistiocytosis; NK cell: Natural killer cell; IL-2: Interleukin-2.

| **The diagnosis HLH can be established if one of either (1) or (2) below is fulfilled** |
| --- |
| **(1) A molecular diagnosis consistent with HLH** |
| **(2) Diagnostic criteria for HLH fulfilled (five out of the eight criteria below)** |
| **(A) Initial diagnostic criteria (to be evaluated in all patients with HLH)** |
| **Fever (≥38.5°C for ≥7 days)** |
| **Splenomegaly** |
| **Cytopenias (affecting 2 of 3 lineages in the peripheral blood)** |
| **Hemoglobin <90 g/L (in infants <4 weeks: hemoglobin <100 g/L)** |
| **Platelets <100*10^9/L**  **Neutrophils <1.0*10^9/L**  **Hypertriglyceridemia and/or hypoﬁbrinogenemia:**  **Fasting triglycerides≥3.0 mmol/L (i.e., 265 mg/dl)**  **Fibrinogen≤1.5 g/L**  **Hemophagocytosis in bone marrow or spleen or lymph nodes (No evidence of malignancy)**  **(B) New diagnostic criteria**  **Low or absent NK cell activity (according to local laboratory reference)**  **Ferritin≥500 mg/L**  **Soluble CD25 (i.e., soluble IL-2 receptor)≥2,400 U/ml** |
