# Supplementary material for: Clinical Value of 18F-FDG PET/CT Scan and Cytokine Profiles in Secondary Hemophagocytic Lymphohistiocytosis in Idiopathic Inflammatory Myopathy Patients: A Pilot Study
Source: Front Immunol. 2021 Nov 18;12:745211. doi: 10.3389/fimmu.2021.745211 (PMC8636988; doi:10.3389/fimmu.2021.745211)
Supplement: Supplementary file 2 [file Table_2.docx]

**Supplementary table 2: Overview of clinical features included in revised diagnostic guideline of HLH within HLH and non-HLH group.**

HLH: Hemophagocytic lymphohistiocytosis; NK cell: Natural killer cell; CD: Clusters of differentiation.

| **Clinical Features** | **HLH Group(10)** | **Non-HLH Group(59)** |
| --- | --- | --- |
| **A molecular diagnosis consistent with HLH** | **NA** | **NA** |
| **Fever (≥38.5°C for ≥7 days)** | **10(100.0%)** | **18(30.5%)** |
| **Splenomegaly** | **9(90.0%)** | **16(27.1%)** |
| **Cytopenias (affecting 2 of 3 lineages in the peripheral blood)** | **10(100.0%)** | **0(0.0%)** |
| Hemoglobin <90 g/L | 5(50.0%) | 1(1.7%) |
| Platelets <100*10^9/L | 10(100.0%) | 5(8.5%) |
| Neutrophils <1.0*10^9/L | 10(100.0%) | 6(6.2%) |
| **Hypertriglyceridemia and/or hypoﬁbrinogenemia** | **10(100.0%)** | **9(15.3%)** |
| Fasting triglycerides≥3.0 mmol/L (i.e., 265 mg/dl) | 7(70.0%) | 7(11.9%) |
| Fibrinogen≤1.5 g/L | 4(40.0%) | 2(3.4%) |
| **Hemophagocytosis in bone marrow** | **5 (50.0%)** | **0(0.0%)** |
| **Low or absent NK cell activity** | **NA** | **NA** |
| **Ferritin≥500 mg/L** | **18(100.0%)** | **35(59.3%)** |
| **Soluble CD25≥2,400 U/ml** | **2 (20.0%)** | **NA** |
