# Supplementary material for: Clinical Value of 18F-FDG PET/CT Scan and Cytokine Profiles in Secondary Hemophagocytic Lymphohistiocytosis in Idiopathic Inflammatory Myopathy Patients: A Pilot Study
Source: Front Immunol. 2021 Nov 18;12:745211. doi: 10.3389/fimmu.2021.745211 (PMC8636988; doi:10.3389/fimmu.2021.745211)
Supplement: Supplementary file 3 [file Table_3.docx]

# Supplementary table 3 Distribution of clinical factors in HLH-2004 diagnostic criteria in ten IIM patients with secondary HLH

● stands for positive. HLH: Haemophagocytic lymphohistiocytosis; IIM: Idiopathic inflammatory myopathy; F: Female; M: Male; NK cell: Natural killer cell; NA: Not available; CD: Clusters of differentiation.

| **Coding** | **1** | **2** | **3** | **4** | **5** | **6** | **7** | **8** | **9** | **10** |
| --- | --- | --- | --- | --- | --- | --- | --- | --- | --- | --- |
| **Age** | **64** | **61** | **43** | **63** | **45** | **42** | **61** | **52** | **64** | **64** |
| **Sex** | **F** | **F** | **F** | **F** | **F** | **M** | **F** | **F** | **F** | **F** |
| **Fever** | **●** | **●** | **●** | **●** | **●** | **●** | **●** | **●** | **●** | **●** |
| **Splenomegaly** |  | **●** | **●** | **●** | **●** | **●** | **●** | **●** | **●** | **●** |
| **Cytopenias** | **●** | **●** | **●** | **●** | **●** | **●** | **●** | **●** | **●** | **●** |
| **Hypertriglyceridemia Hypoﬁbrinogenemia** | **●** | **●** | **●** | **●** | **●** | **●** | **●** | **●** | **●** | **●** |
| **Hemophagocytosis in bone marrow** | **●** | **●** | **●** |  | **●** |  | **●** |  |  |  |
| **Ferritin≥500 mg/L** | **●** | **●** | **●** | **●** | **●** | **●** | **●** | **●** | **●** | **●** |
| **Low or absent NK cell activity** | **NA** | **NA** | **NA** | **NA** | **NA** | **NA** | **NA** | **NA** | **NA** | **NA** |
| **Soluble CD25 ≥2,400 U/ml** | **NA** | **NA** | **●** | **NA** | **●** | **NA** | **NA** | **NA** | **NA** | **NA** |
