# Supplementary material for: Clinical Value of 18F-FDG PET/CT Scan and Cytokine Profiles in Secondary Hemophagocytic Lymphohistiocytosis in Idiopathic Inflammatory Myopathy Patients: A Pilot Study
Source: Front Immunol. 2021 Nov 18;12:745211. doi: 10.3389/fimmu.2021.745211 (PMC8636988; doi:10.3389/fimmu.2021.745211)
Supplement: Supplementary file 4 [file Table_4.docx]

**Supplementary table 4 Summary of the causes of death in HLH and non-HLH groups**

HLH: Haemophagocytic lymphohistiocytosis; RP-ILD: Rapidly progressive interstitial lung disease; DIC: Disseminated intravascular coagulation; ILD: Interstitial lung disease.

| **HLH group** | | **Non-HLH group** | |
| --- | --- | --- | --- |
| **Cause of death** | **8 (100%)** | **Cause of death** | **23 (100%)** |
| **Infection** | **2(25.0%)** | **Infection** | **1(4.3%)** |
| **Infection and HLH** | **2(25.0%)** | **Infection and RP-ILD** | **3(13.0%)** |
| **RP-ILD** | **1(12.5%)** | **RP-ILD** | **2(8.7%)** |
| **RP-ILD and heart failure** | **1(12.5%)** | **RP-ILD and heart failure** | **1(4.3%)** |
| **DIC** | **1(12.5%)** | **ILD exacerbation (not satisfying RP-ILD)** | **3(13.0%)** |
| **Aspiration** | **1(12.5%)** | **Pulmonary hypertension** | **1(4.3%)** |
|  |  | **Aspiration** | **1(4.3%)** |
|  |  | **Carcinoma** | **5(21.7%)** |
|  |  | **Gastrointestinal bleeding** | **1(4.3%)** |
|  |  | **Ischemic stroke** | **2(8.7%)** |
|  |  | **Intracerebral hemorrahge** | **1(4.3%)** |
|  |  | **Ischemic heart disease** | **2(8.7%)** |
|  |  |  |  |
|  |  |  |  |
