# Supplementary material for: Clinical Value of 18F-FDG PET/CT Scan and Cytokine Profiles in Secondary Hemophagocytic Lymphohistiocytosis in Idiopathic Inflammatory Myopathy Patients: A Pilot Study
Source: Front Immunol. 2021 Nov 18;12:745211. doi: 10.3389/fimmu.2021.745211 (PMC8636988; doi:10.3389/fimmu.2021.745211)
Supplement: Supplementary file 5 [file Table_5.docx]

**Supplementary table 5 Summary of immunosuppressive details for IIM patients who developed sHLH**

IIM: Idiopathic inflammatory myopathy; sHLH: Secondary haemophagocytic lymphohistiocytosis; IVIG: Intravenous immunoglobulin.

*Some of the included patients were not diagnosed with sHLH during hospitalization, the diagnosis was verified when retrospectively reviewing these patients’ medical records.

| **Coding** | **Immunosuppressive medications** |
| --- | --- |
| **1** | **Steroid** |
| **2** | **Etoposide, Steroid, IVIG** |
| **3** | **Steroid, IVIG** |
| **4** | **Steroid** |
| **5** | **Etoposide, Steroid, IVIG** |
| **6** | **Steroid, Cyclosporine** |
| **7** | **Steroid, Tacrolimus, Hydroxychloroquine** |
| **8** | **Etoposide, Steroid, Thalidomide** |
| **9** | **Steroid Cyclosporine** |
| **10** | **Steroid** |
