# Supplementary material for: Clinical Value of 18F-FDG PET/CT Scan and Cytokine Profiles in Secondary Hemophagocytic Lymphohistiocytosis in Idiopathic Inflammatory Myopathy Patients: A Pilot Study
Source: Front Immunol. 2021 Nov 18;12:745211. doi: 10.3389/fimmu.2021.745211 (PMC8636988; doi:10.3389/fimmu.2021.745211)
Supplement: Supplementary file 6 [file Table_6.docx]

# Supplementary table 6 Univariate logistic regression analyses for sHLH in IIM patients

sHLH: Secondary haemophagocytic lymphohistiocytosis; IIM: Idiopathic inflammatory myopathy; OR: Odds ratio; CI: Confidence interval; P-adjusted: Adjusted P value after false discovery rate correction; y: years; EBV: Epstein-Barr virus; CMV: Cytomegalovirus; RP-ILD: Rapidly progressive interstitial lung disease; MYOACT: Myositis Disease Activity Assessment Visual Analogue Scales; CD: Clusters of differentiation; IL: Interleukin; TNF: Tumor necrosis factor; IFN: Interferon; CRP: C-reactive protein; ESR: Erythrocyte sedimentation rate; ALT: Alaninetransaminase; AST: Aspartate transaminase; LDH: Lactate dehydrogenase; CK: Creatine kinase; FDG: Fluorodeoxyglucose; SUVmean: Mean standard uptake value; NA: Not available; DMARDs*:* Disease-modifying anti-rheumatic drugs; IVIG: Intravenous immunoglobulin; JAK: Janus kinase; IIM: Idiopathic inflammatory myopathy; DM: dermatomyositis; PM: Polymyositis; ADM: Amyopathic dermatomyositis.

| **Factors** | **P value** | **OR value** | **95%CI** | **P-adjusted** |
| --- | --- | --- | --- | --- |
| **Age(y)** | **0.855** | **0.995** | **0.939~1.053** | **1.000** |
| **Sex (male/female)** | **0.062** | **0.132** | **0.016~1.106** | **0.234** |
| **Clinical manifestations or complications** | | | |  |
| **Pulmonary bacterial infection** | **0.007** | **7.429** | **1.709~32.290** | **0.078** |
| **Pulmonary fungal infection** | **0.485** | **1.857** | **0.326~10.569** | **0.942** |
| **Tuberculosis infection** | **1.000** | **<0.001** | **<0.001~>100.000** | **1.000** |
| **EBV infection** | **0.396** | **0.393** | **0.046~3.395** | **0.869** |
| **CMV infection** | **0.999** | **<0.001** | **<0.001~>100.000** | **1.000** |
| **RP-ILD** | **0.480** | **1.647** | **0.412~6.579** | **0.942** |
| **Carcinoma** | **0.999** | **<0.001** | **<0.001~>100.000** | **1.000** |
| **Disease activity** | | | |  |
| **MYOACT score** | **0.012** | **1.271** | **1.055~1.531** | **0.091** |
| **Laboratory finding** | | | |  |
| **CD3^+^CD4^+^ lymphocytes (%)** | **0.393** | **0.978** | **0.928~1.030** | **0.869** |
| **CD3^+^CD8^+^ lymphocytes (%)** | **0.054** | **1.046** | **0.999~1.094** | **0.232** |
| **CD4^+^/ CD8^+^ Ratio** | **0.146** | **0.575** | **0.273~1.212** | **0.496** |
| **CD3^-^CD16^+^CD56^+^ lymphocytes(%)** | **0.040** | **0.845** | **0.720~0.993** | **0.232** |
| **CD3^-^CD19^+^ lymphocytes(%)** | **0.670** | **1.012** | **0.957~1.071** | **1.000** |
| **IL-2(pg/ml)** | **0.058** | **1.194** | **0.994~1.434** | **0.232** |
| **IL-4(pg/ml)** | **0.002** | **2.451** | **1.393~4.311** | **0.045** |
| **IL-6(pg/ml)** | **0.007** | **1.018** | **1.005~1.031** | **0.078** |
| **IL-10(pg/ml)** | **0.049** | **1.010** | **1.000~1.021** | **0.232** |
| **TNF-α(pg/ml)** | **0.003** | **1.530** | **1.161~2.016** | **0.051** |
| **IFN-γ(pg/ml)** | **0.001** | **1.437** | **1.167~1.769** | **0.034** |
| **IL-17A(pg/ml)** | **0.887** | **0.997** | **0.963~1.033** | **1.000** |
| **CRP(mg/L)** | **0.262** | **0.974** | **0.931~1.020** | **0.742** |
| **ESR(mm/h)** | **0.349** | **1.011** | **0.989~1.033** | **0.858** |
| **ALT(U/L)** | **0.057** | **1.003** | **1.000~1.006** | **0.232** |
| **AST(U/L)** | **0.011** | **1.003** | **1.001~1.005** | **0.091** |
| **LDH(U/L)** | **0.688** | **1.000** | **0.998~1.003** | **1.000** |
| **CK(U/L)** | **0.551** | **1.000** | **0.999~1.000** | **1.000** |
| **^18^F-FDG PET/CT scan findings** | | | | |
| **Bilateral lung SUVmean** | **0.045** | **59.385** | **1.099~>100.000** | **0.232** |
| **Liver SUVmean** | **0.363** | **0.462** | **0.088~2.437** | **0.858** |
| **Spleen SUVmean** | **0.001** | **48.822** | **4.665~>100.000** | **0.034** |
| **Bone marrow SUVmean** | **0.134** | **2.945** | **0.716~12.112** | **0.480** |
| **Cardiac SUVmean** | **0.192** | **0.548** | **0.222~1.353** | **0.593** |
| **Esophagus SUVmean** | **0.987** | **0.990** | **0.299~3.279** | **1.000** |
| **Stomach SUVmean** | **0.165** | **3.211** | **0.618~16.677** | **0.534** |
| **Small intestine SUVmean** | **0.794** | **1.332** | **0.154~11.504** | **1.000** |
| **Colon and rectum SUVmean** | **0.042** | **3.609** | **1.048~12.428** | **0.232** |
| **Bilateral cerebellum SUVmean** | **0.942** | **1.016** | **0.657~1.572** | **1.000** |
| **Bilateral trapezius SUVmean** | **0.226** | **0.179** | **0.011~2.898** | **0.668** |
| **Bilateral deltoid SUVmean** | **0.321** | **0.330** | **0.037~2.942** | **0.858** |
| **Bilateral biceps SUVmean** | **0.440** | **0.408** | **0.042~3.964** | **0.907** |
| **Bilateral ilioposas SUVmean** | **0.524** | **1.816** | **0.290~11.377** | **0.990** |
| **Bilateral gluteus maximus SUVmean** | **0.743** | **1.323** | **0.249~7.029** | **1.000** |
| **Bilateral gluteus medius SUVmean** | **0.359** | **0.317** | **0.027~3.689** | **0.858** |
| **Bilateral quadriceps SUVmean** | **0.712** | **0.651** | **0.067~6.334** | **1.000** |
| **Myositis-specific antibodies & Myositis-associated antibodies** | | | |  |
| **Anti-MDA5** | **0.008** | **9.111** | **1.757~47.233** | **0.078** |
| **Anti-PL-7** | **0.987** | **0.981** | **0.105~9.144** | **0.987** |
| **Anti-PL-12** | **0.999** | **<0.001** | **<0.001~>100.000** | **1.000** |
| **Anti-EJ** | **0.999** | **<0.001** | **<0.001~>100.000** | **1.000** |
| **Anti-OJ** | **1.000** | **<0.001** | **<0.001~>100.000** | **1.000** |
| **Anti-Jo-1** | **0.999** | **<0.001** | **<0.001~>100.000** | **1.000** |
| **Anti-TIF1γ** | **0.999** | **<0.001** | **<0.001~>100.000** | **1.000** |
| **Anti-Mi-2α** | **0.999** | **<0.001** | **<0.001~>100.000** | **1.000** |
| **Anti-Mi-2β** | **0.999** | **<0.001** | **<0.001~>100.000** | **1.000** |
| **Anti-SAE1** | **0.999** | **<0.001** | **<0.001~>100.000** | **1.000** |
| **Anti-NXP2** | **0.999** | **<0.001** | **<0.001~>100.000** | **1.000** |
| **Anti-SRP** | **0.366** | **3.167** | **0.260~38.627** | **0.858** |
| **Anti-Ku** | **0.999** | **<0.001** | **<0.001~>100.000** | **1.000** |
| **Anti-PM-Scl75** | **0.999** | **<0.001** | **<0.001~>100.000** | **1.000** |
| **Anti-PM-Scl100** | **NA** | **NA** | **NA** | **NA** |
| **Anti-Ro-52** | **0.409** | **1.778** | **0.454~1.961** | **0.869** |
| **Therapies** | | | |  |
| **Steroid monotherapy** | **0.809** | **0.836** | **0.195~3.584** | **1.000** |
| **Steroid+DMARDs** | **0.968** | **0.972** | **0.248~3.817** | **1.000** |
| [**Steroid+IVIG**](http://www.baidu.com/link?url=_srwKTXKnet8GknUvvs0xyTJdpfNOQtIDWHWhe_U5wypEldT9OPh2gCg3LsSDR-5CpyLTLOBAy4p4ov8wle8F6_YWPs4sPX-lyXINgDKaDW) | **0.040** | **5.893** | **1.086~31.968** | **0.232** |
| [**Steroid+DMARDs+IVIG**](http://www.baidu.com/link?url=uciYHxddnq2QF5VJVWJRCy7Q7nEAXlzzmiKvgGzZkrPg72XHW0qrc1acnFRmU-CtSPSZqd_rW-WBKuZFe0OpuS_h9gOsjyItDqvwfb_UtbdGjXJvU0FWCCPVF1qaXYLk) | **0.999** | **<0.001** | **<0.001~>100.000** | **1.000** |
| **Steroid+JAK inhibitor** | **0.999** | **<0.001** | **<0.001~>100.000** | **1.000** |
| **IIM subtypes** | | | |  |
| **DM** | **0.708** | **0.769** | **0.194~3.043** | **1.000** |
| **PM** | **0.814** | **1.225** | **0.226~6.653** | **1.000** |
| **ADM** | **0.814** | **1.225** | **0.226~6.653** | **1.000** |
