# Supplementary material for: Clinical Value of 18F-FDG PET/CT Scan and Cytokine Profiles in Secondary Hemophagocytic Lymphohistiocytosis in Idiopathic Inflammatory Myopathy Patients: A Pilot Study
Source: Front Immunol. 2021 Nov 18;12:745211. doi: 10.3389/fimmu.2021.745211 (PMC8636988; doi:10.3389/fimmu.2021.745211)
Supplement: Supplementary file 7 [file Table_7.docx]

# Supplementary table 7 Multivariate logistic regression analysis for sHLH in IIM patients

# sHLH: Secondary haemophagocytic lymphohistiocytosis; IIM: Idiopathic inflammatory myopathy; OR: Odds ratio; CI: Confidence interval; IFN: Interferon; SUVmean: Mean standard uptake value.

| **Factors** | **P value** | **OR value** | **95%CI** |
| --- | --- | --- | --- |
| **IFN-γ(pg/ml)** | **0.017** | **1.636** | **1.093~2.448** |
| **Spleen SUVmean** | **0.035** | **23.418** | **1.240~>100.000** |
| **Anti-MDA5 antibody** | **0.049** | **18.967** | **1.011~>100.000** |
