# Supplementary material for: Clinical Value of 18F-FDG PET/CT Scan and Cytokine Profiles in Secondary Hemophagocytic Lymphohistiocytosis in Idiopathic Inflammatory Myopathy Patients: A Pilot Study
Source: Front Immunol. 2021 Nov 18;12:745211. doi: 10.3389/fimmu.2021.745211 (PMC8636988; doi:10.3389/fimmu.2021.745211)
Supplement: Supplementary file 8 [file Table_8.docx]

# Supplementary table 8 Comparisons in IIM patients (with sHLH) who died within three months or survived beyond this threshold

IIM: Idiopathic inflammatory myopathy; sHLH: Secondary haemophagocytic lymphohistiocytosis; P-adjusted: Adjusted P value after false discovery rate correction; y: years; NA: Not available; EBV: Epstein-Barr virus; CMV: Cytomegalovirus; RP-ILD: Rapidly progressive interstitial lung disease; MYOACT: Myositis Disease Activity Assessment Visual Analogue Scales; CD: Clusters of differentiation; IL: Interleukin; TNF: Tumor necrosis factor; IFN: Interferon; CRP: C-reactive protein; ESR: Erythrocyte sedimentation rate; ALT: Alaninetransaminase; AST: Aspartate transaminase; LDH: Lactate dehydrogenase; CK: Creatine kinase; FDG: Fluorodeoxyglucose; SUVmean: mean standard uptake value; DMARDs*:* Disease-modifying anti-rheumatic drugs; IVIG: Intravenous immunoglobulin; JAK: Janus kinase; DM: dermatomyositis; PM: Polymyositis; ADM: Amyopathic dermatomyositis.

| **Factors** | **<3 months (6)** | **>3 months (4)** | **P value** | **P-adjusted** |
| --- | --- | --- | --- | --- |
| **Age(y)** | **48.50(42.75, 61.50)** | **64.00 (61.75, 64.00)** | **0.019** | **0.494** |
| **Sex(male/female)** | **1/5** | **0/4** | **1.000** | **1.000** |
| **Clinical manifestations or complications** | | | |  |
| **Pulmonary bacterial infection** | **3(50.0%)** | **2(50.0%)** | **1.000** | **1.000** |
| **Pulmonary fungal infection** | **1(16.7%)** | **1(25.0%)** | **1.000** | **1.000** |
| **Tuberculosis infection** | **0(0.0%)** | **0(0.0%)** | **NA** | **NA** |
| **EBV infection** | **1(16.7%)** | **0(0.0%)** | **1.000** | **1.000** |
| **CMV infection** | **0(0.0%)** | **0(0.0%)** | **NA** | **NA** |
| **RP-ILD** | **3(50.0%)** | **1(25.0%)** | **0.571** | **0.928** |
| **Carcinoma** | **0(0.0%)** | **0(0.0%)** | **NA** | **NA** |
| **Disease activity** | | | |  |
| **MYOACT score** | **15.50 (12.50, 17.25)** | **10.50(6.75, 15.00)** | **0.171** | **0.593** |
| **Laboratory finding** | | | |  |
| **CD3^+^CD4^+^ lymphocytes (%)** | **41.66(31.55,54.58)** | **27.05 (16.43, 39.55)** | **0.257** | **0.703** |
| **CD3^+^CD8^+^ lymphocytes (%)** | **22.55(16.70,32.55)** | **39.15 (28.28, 69.00)** | **0.114** | **0.593** |
| **CD4^+^/ CD8^+^ Ratio** | **1.96(1.05, 2.80)** | **0.80 (0.25, 1.40)** | **0.114** | **0.593** |
| **CD3^-^CD16^+^CD56^+^ lymphocytes(%)** | **5.20(1.35, 10.28)** | **7.90 (5.30, 9.68)** | **0.762** | **0.991** |
| **CD3^-^CD19^+^ lymphocytes(%)** | **22.60(13.10, 29.85)** | **22.30 (5.03, 27.73)** | **1.000** | **1.000** |
| **IL-2(pg/ml)** | **0.68(0.10,7.48)** | **3.79 (0.10, 7.95)** | **0.914** | **1.000** |
| **IL-4(pg/ml)** | **2.23(0.10,3.78)** | **3.88 (1.13, 6.52)** | **0.257** | **0.703** |
| **IL-6(pg/ml)** | **45.01(16.10,165.74)** | **35.25 (6.44, 92.23)** | **0.762** | **0.991** |
| **IL-10(pg/ml)** | **19.32(14.91,164.71)** | **13.33 (3.81, 24.70)** | **0.476** | **0.825** |
| **TNF-α(pg/ml)** | **10.19(4.64,34.73)** | **4.94 (0.49, 18.59)** | **0.352** | **0.796** |
| **IFN-γ(pg/ml)** | **7.25(4.00,17.44)** | **10.69 (2.35, 15.01)** | **1.000** | **1.000** |
| **IL-17A(pg/ml)** | **3.68(0.10, 28.81)** | **1.20 (0.10, 9.16)** | **0.476** | **0.825** |
| **CRP(mg/L)** | **3.10(0.93, 5.78)** | **5.80 (1.78, 42.35)** | **0.476** | **0.825** |
| **ESR(mm/h)** | **14.00(7.50, 42.50)** | **54.00 (17.75, 81.25)** | **0.114** | **0.593** |
| **ALT(U/L)** | **126.50(58.75, 637.50)** | **134.00 (106.00, 241.50)** | **0.914** | **1.000** |
| **AST(U/L)** | **268.50(89.75, 1091.25)** | **136.00 (48.50, 717.00)** | **0.352** | **0.796** |
| **LDH(U/L)** | **353.00(222.50, 738.75)** | **417.00 (323.00, 581.50)** | **0.610** | **0.933** |
| **CK(U/L)** | **255.00(100.75, 1359.50)** | **64.50 (31.50, 84.75)** | **0.038** | **0.593** |
| **^18^F-FDG PET/CT scan findings** | | | | |
| **Bilateral lung SUVmean** | **0.55(0.35, 0.82)** | **0.62 (0.43, 0.69)** | **0.762** | **0.991** |
| **Liver SUVmean** | **1.79(1.43, 1.92)** | **1.33 (1.27, 1.66)** | **0.171** | **0.593** |
| **Spleen SUVmean** | **2.25(2.07, 3.14)** | **2.37 (2.20, 2.60)** | **0.610** | **0.933** |
| **Bone marrow SUVmean** | **2.02(1.64, 2.43)** | **1.44 (1.04, 2.21)** | **0.352** | **0.796** |
| **Cardiac SUVmean** | **1.40(0.92, 2.01)** | **1.24 (1.13, 1.37)** | **0.762** | **0.991** |
| **Esophagus SUVmean** | **1.40(1.17, 2.04)** | **1.06 (0.87, 1.61)** | **0.171** | **0.593** |
| **Stomach SUVmean** | **0.80(0.51, 1.45)** | **0.74 (0.48, 1.20)** | **1.000** | **1.000** |
| **Small intestine SUVmean** | **1.12 (1.03, 1.32)** | **0.84 (0.78, 1.22)** | **0.476** | **0.825** |
| **Colon and rectum SUVmean** | **1.32(1.22, 2.09)** | **1.15 (0.78, 1.48)** | **0.352** | **0.796** |
| **Bilateral cerebellum SUVmean** | **5.66(4.01, 7.62)** | **4.10 (3.07, 5.58)** | **0.171** | **0.593** |
| **Bilateral trapezius SUVmean** | **0.79(0.66, 1.05)** | **0.68 (0.56, 0.70)** | **0.114** | **0.593** |
| **Bilateral deltoid SUVmean** | **0.75(0.68, 0.94)** | **0.72 (0.47, 0.88)** | **0.762** | **0.991** |
| **Bilateral biceps SUVmean** | **0.83(0.59, 1.04)** | **0.79 (0.70, 0.95)** | **1.000** | **1.000** |
| **Bilateral ilioposas SUVmean** | **1.25(1.07, 1.46)** | **1.07 (0.65, 1.69)** | **0.762** | **0.991** |
| **Bilateral gluteus maximus SUVmean** | **0.77(0.59, 1.06)** | **0.79 (0.64, 1.35)** | **0.914** | **1.000** |
| **Bilateral gluteus medius SUVmean** | **0.95(0.82, 1.07)** | **0.79 (0.66, 0.88)** | **0.114** | **0.593** |
| **Bilateral quadriceps SUVmean** | **0.88(0.70, 1.20)** | **0.77 (0.62, 0.83)** | **0.476** | **0.825** |
| **Myositis-specific antibodies & Myositis-associated antibodies** | | | |  |
| **Anti-MDA5** | **6(100.0%)** | **2(50.0%)** | **0.133** | **0.593** |
| **Anti-PL-7** | **0(0.0%)** | **1(25.0%)** | **0.400** | **0.825** |
| **Anti-PL-12** | **0(0.0%)** | **0(0.0%)** | **NA** | **NA** |
| **Anti-EJ** | **0(0.0%)** | **0(0.0%)** | **NA** | **NA** |
| **Anti-OJ** | **0(0.0%)** | **0(0.0%)** | **NA** | **NA** |
| **Anti-Jo-1** | **0(0.0%)** | **0(0.0%)** | **NA** | **NA** |
| **Anti-TIF1γ** | **0(0.0%)** | **0(0.0%)** | **NA** | **NA** |
| **Anti-Mi-2α** | **0(0.0%)** | **0(0.0%)** | **NA** | **NA** |
| **Anti-Mi-2β** | **0(0.0%)** | **0(0.0%)** | **NA** | **NA** |
| **Anti-SAE1** | **0(0.0%)** | **0(0.0%)** | **NA** | **NA** |
| **Anti-NXP2** | **0(0.0%)** | **0(0.0%)** | **NA** | **NA** |
| **Anti-SRP** | **0(0.0%)** | **1(25.0%)** | **0.400** | **0.825** |
| **Anti-Ku** | **0(0.0%)** | **0(0.0%)** | **NA** | **NA** |
| **Anti-PM-Scl75** | **0(0.0%)** | **0(0.0%)** | **NA** | **NA** |
| **Anti-PM-Scl100** | **0(0.0%)** | **0(0.0%)** | **NA** | **NA** |
| **Anti-Ro-52** | **5(83.3%)** | **1(25.0%)** | **0.190** | **0.612** |
| **Therapies** | | | |  |
| **Steroid monotherapy** | **1(16.7%)** | **2(50.0%)** | **0.500** | **0.839** |
| **Steroid+DMARDs** | **2(33.3%)** | **2(50.0%)** | **1.000** | **1.000** |
| [**Steroid+IVIG**](http://www.baidu.com/link?url=_srwKTXKnet8GknUvvs0xyTJdpfNOQtIDWHWhe_U5wypEldT9OPh2gCg3LsSDR-5CpyLTLOBAy4p4ov8wle8F6_YWPs4sPX-lyXINgDKaDW) | **3(50.0%)** | **0(0.0%)** | **0.200** | **0.612** |
| [**Steroid+DMARDs+IVIG**](http://www.baidu.com/link?url=uciYHxddnq2QF5VJVWJRCy7Q7nEAXlzzmiKvgGzZkrPg72XHW0qrc1acnFRmU-CtSPSZqd_rW-WBKuZFe0OpuS_h9gOsjyItDqvwfb_UtbdGjXJvU0FWCCPVF1qaXYLk) | **0(0.0%)** | **0(0.0%)** | **NA** | **NA** |
| **Steroid+JAK inhibitor** | **0(0.0%)** | **0(0.0%)** | **NA** | **NA** |
| **IIM subtypes** | | | |  |
| **DM** | **6(100.0%)** | **0(0.0%)** | **0.005** | **0.260** |
| **PM** | **0(0.0%)** | **2(50.0%)** | **0.133** | **0.593** |
| **ADM** | **0(0.0%)** | **2(50.0%)** | **0.133** | **0.593** |
