# Supplementary figures and images for: Clinical Value of 18F-FDG PET/CT Scan and Cytokine Profiles in Secondary Hemophagocytic Lymphohistiocytosis in Idiopathic Inflammatory Myopathy Patients: A Pilot Study
Source: Front Immunol. 2021 Nov 18;12:745211. doi: 10.3389/fimmu.2021.745211 (PMC8636988; doi:10.3389/fimmu.2021.745211)

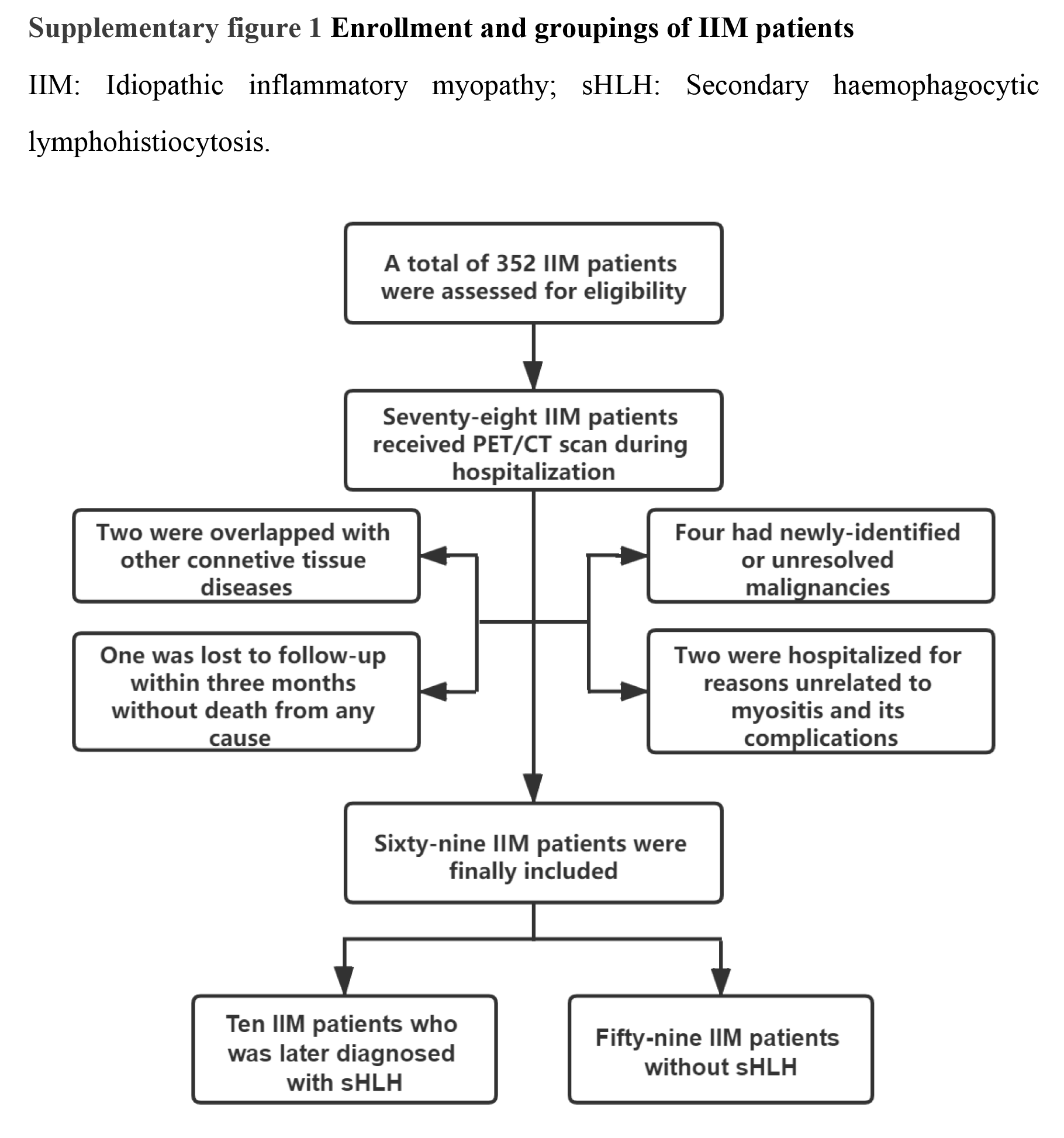

Supplement: Supplementary file 10 [file Image_1.tif]
